# Pyrethroid resistance in the major malaria vector *Anopheles arabiensis* in Nouakchott, Mauritania

**DOI:** 10.1186/s13071-018-2923-4

**Published:** 2018-06-13

**Authors:** Aichetou Mint Mohamed Lemine, Mohamed Aly Ould Lemrabott, El Hadji Amadou Niang, Leonardo K. Basco, Hervé Bogreau, Ousmane Faye, Ali Ould Mohamed Salem Boukhary

**Affiliations:** 1 Unité de recherche Génomes et Milieux (JEAI), Université de Nouakchott Al-Aasriya, Faculté des Sciences et Techniques, Nouveau Campus Universitaire, Nouakchott, BP 5026 Mauritanie; 2 Laboratoire d’Ecologie Vectorielle et Parasitaire, Faculté des Sciences et Techniques, Université Cheikh Anta Diop, Dakar, Sénégal; 3 Aix Marseille Univ, IRD, AP-HM, MEPHI, IHU-Méditerranée Infection, Marseille, France; 4 Aix Marseille Univ, IRD, AP-HM, SSA, VITROME, IHU-Méditerranée Infection, Marseille, France; 5 Institut de Recherche Biomédicale des Armées, Unité de Parasitologie et d’Entomologie, IHU-Méditerranée Infection, Marseille, France

**Keywords:** Pyrethroids, Insecticides, Resistance, *kdr*, *Anopheles arabiensis*, Nouakchott, Mauritania

## Abstract

**Background:**

Mauritania is one of the African countries with ongoing malaria transmission where data on insecticide resistance of local malaria vectors are limited despite an increasing use of long-lasting insecticide-treated nets (LLINs) as the main intervention for vector control. This study presents an evaluation of the level of insecticide resistance of *Anopheles arabiensis* in Nouakchott.

**Methods:**

*Anopheles gambiae* (*s.l.*) larvae were collected in breeding sites during the rainy season (August-September) in 2015 and 2016 from two selected sites in Nouakchott and reared until emergence. Adult anopheline mosquitoes were tested against malathion (5%), bendiocarb (0.1%), permethrin (0.75%) and deltamethrin (0.05%) using standard World Health Organization (WHO) insecticide-impregnated papers. PCR assays were used for the identification of *An. gambiae* (*s.l.*) sibling species as well as knockdown resistance (*kdr*).

**Results:**

The mean knockdown times 50% (KDT_50_) and 95% (KDT_95_) were 66 ± 17 and 244 ± 13 min, respectively, for permethrin in 2015. The KDT_50_ and the KDT_95_ were 39 ± 13 and 119 ± 13 min, respectively, for deltamethrin. The KDT_50_ and the KDT_95_ doubled for both molecules in 2016. The mortality rates 24 h post-exposure revealed that *An. arabiensis* populations in Nouakchott were fully susceptible to bendiocarb and malathion in 2015 as well as in 2016, while they were resistant to permethrin (51.9% mortality in 2015 and 24.1% mortality in 2016) and to deltamethrin (83.7% mortality in 2015 and 39.1% mortality in 2016). The molecular identification showed that *Anopheles arabiensis* was the only malaria vector species collected in Nouakchott in 2015 and 2016. Both the West and East African *kdr* mutant alleles were found in *An. arabiensis* mosquitoes surviving exposure to pyrethroid insecticide, with a high rate of homozygous resistant genotypes (54.3% for the West African *kdr* mutation and 21.4% for the East African *kdr* mutation) and a significant departure from Hardy-Weinberg proportions (*χ*^2^ = 134, *df* = 3, *P* < 0.001).

**Conclusions:**

The study showed high levels of pyrethroid resistance in *An. arabiensis* populations in Nouakchott and presence of both West and East African *kdr* alleles in the resistant phenotype. These results highlight a need for routine monitoring of susceptibility of malaria vector populations to insecticides used in public health programs.

## Background

Long-lasting insecticide-treated nets (LLINs) and indoor residual spraying (IRS), the most commonly used malaria vector control interventions, rely heavily on synthetic insecticides [1]. Currently, four chemical classes of insecticides are recommended by the World Health Organization Pesticide Evaluation Scheme (WHOPES) for use in public health programs: organochlorines, organophosphates, pyrethroids and carbamates [2]. Among these, pyrethroids are by far the most frequently used insecticides due to their relatively low toxicity to humans, fast knockdown effect and cost-effectiveness [3]. Moreover, pyrethroids are the only insecticides approved by the World Health Organization (WHO) for net impregnation [4]. Extensive use of insecticide-based interventions has led to massive reduction of malaria burden in many parts of Africa [5]. However, this strategy has subjected malaria vectors, in particular *An. gambiae* (*sensu lato*) which is the predominant *Anopheles* species in Africa, to selection pressures [6].

In Africa, resistance to DDT was first reported in 1967 in *An. gambiae* (*s.l.*) populations from Burkina Faso and hampered malaria eradication or control efforts led by the WHO since the 1950s. *Anopheles funestus*, the second major malaria vector in Africa, has also developed resistance to several insecticides in many parts of the African continent [6]. More recently, insecticide resistance in other vector species has been reported from many other countries [7]. According to the WHO, resistance to at least one insecticide class has been recorded in at least one anopheline species from nearly two-thirds of the countries with ongoing malaria transmission [8, 9]. This continual selection of insecticide resistance and its geographical spread are threatening the gains made against the disease worldwide. Like the insecticide resistance among vectors, the resurgence of malaria and drug-resistant malaria parasites is a serious threat to current malaria control strategies and efforts to achieve the malaria elimination goal [10, 11]. Pyrethroids have become the mainstay of mosquito control. Pyrethroids are neurotoxic chemicals that cause prolonged opening of the voltage-gated sodium channel of the neuron membrane, leading to increased nerve impulse transmission, paralysis, and eventual death of the insect [12]. Specific mutations in the gene coding for the voltage-gated sodium channel decrease neuronal sensitivity to pyrethroids, resulting in insecticide resistance [13]. This form of resistance, known as knockdown resistance (*kdr*), is one of the most common mechanisms of resistance among insects, including anophelines [14–16]. Two point mutations occurring in codon 1014 of the voltage-gated para sodium channel gene have been associated with insecticide resistance in *Anopheles gambiae* (*s.l.*) in Africa [17]. These mutations result in either a leucine to phenylalanine (L1014F) substitution, referred to as West African *kdr* mutation [18], or a leucine to serine substitution (L1014S), known as East African *kdr* mutation [19, 20].

In Mauritania, malaria transmission is seasonal in most regions of the country [21, 22]. *Plasmodium falciparum* is the predominant malaria parasite in the southern Sahelian zone of the country, while *Plasmodium vivax* is more prevalent than *P. falciparum* in the northern Saharan zone [23–26]. A recent literature review indicated that 17 *Anopheles* species have been described throughout the country, among which *An. gambiae* (*s.l.*) is the most common and widespread species [27, 28]. Molecular identification of the *An. gambiae* complex showed that *An. arabiensis* is the main anopheline species in the country. Mint Lekweiry et al. [28] found only *An. arabiensis* in Nouakchott, the capital city of Mauritania that is located in the western Saharan region of the country. In the southeastern Sahelian zone of Hodh Elgharbi, this species accounted for only 40% of all the mosquitoes collected [28]. However, earlier studies have reported the predominance of *An. arabiensis* (97%, *n* = 448) in Hodh Elgharbi (SF Traoré, unpublished WHO report, 2002). The presence of *Anopheles coluzzii* Coetzee & Wilkerson (previously M form), another member of the *An. gambiae* complex, was also reported (3%, *n* = 13), but only in the Sahelian regions of Boghé and Aioun.

Mauritania is one of the African countries with ongoing malaria transmission where the resistance status of local malaria vectors to different insecticide classes is not well characterized [29]. The first study conducted in the Sahelian region of the country in 2002 reported a full susceptibility of *An. arabiensis* to permethrin in Rosso, Boghe, Selibaby and Aioun and also full susceptibility of *An. pharoensis* to deltamethrin in Rosso and Boghe (Traore SF, unpublished WHO report, 2002). In 2009–2010, the presence of both the West and East African *kdr* mutant alleles was first reported in *An. gambiae* (*s.l.*) populations in Mauritania [28].

The official published data reported the distribution of 165,000 LLINs in Mauritania from 2004 to 2005 [30]. In 2016, over 200,000 LLINs were distributed nationwide, thus substantially increasing bed-net ownership throughout the country. It is now estimated that more than 60% of the population owns at least one bednet (National Malaria Control Program, unpublished data). Therefore, with the scaling up of the distribution and increasing use of LLINs in Mauritania as well as the widespread occurrence of pyrethroid resistance in malaria vectors reported from several West and East African countries, there is an urgent need to establish the current status of insecticide resistance of the main malaria vectors in the country and assess the impact of insecticide-based vector control interventions [28, 31].

In the present study, we assessed the resistance status of *An. arabiensis* populations collected from two districts in Nouakchott to four insecticides used in public health programs. This data fills a gap in our knowledge and will improve current and future vector control strategies in Mauritania.

## Methods

### Study sites and mosquito collection

**Fig. 1:**
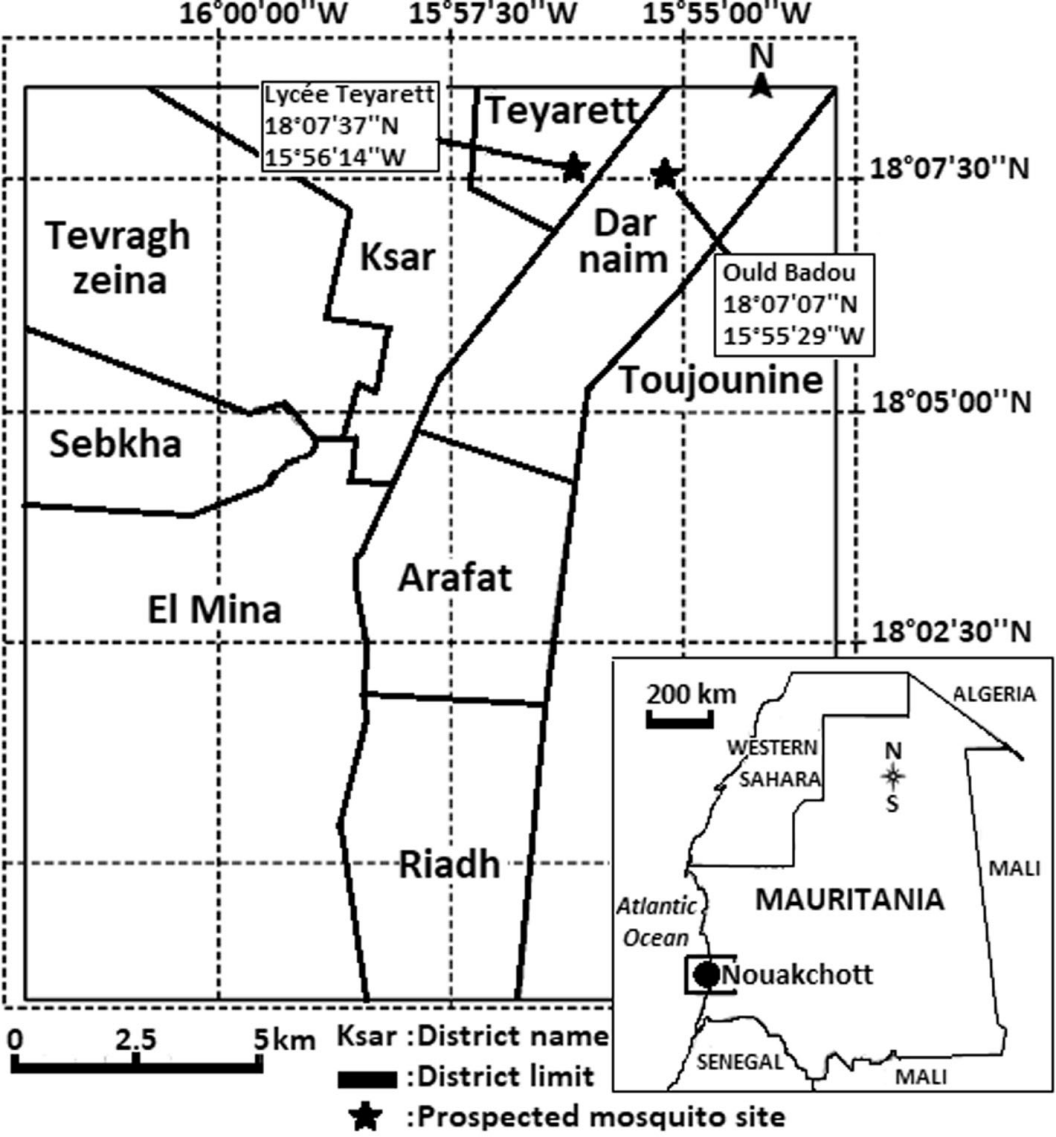
Map showing the study sites in Nouakchott. Inset: map of Mauritania

Entomological surveys were carried out during the wet season (August-September) in 2015 and 2016 in two selected sites in Nouakchott: Lycée de Teyarett (18°07'37"N, 15°56'14"W) and Carrefour Ould Badou (18°07'07"N, 15°55'29"W) (Fig. 1). In these areas, breeding sites mainly consisted of water discharged from public standpipes. The physical and chemical characteristics of the habitats of *An. gambiae* (*s.l.*) in Nouakchott are published elsewhere [31].

In each study site, two larval habitats were sampled. Larvae and pupae of *Anopheles* mosquito were collected using the “dipping” sampling method and reared in the insectarium to adulthood. Larvae were kept in separate labelled bottles and transported to the insectary. *Anopheles* larvae were separated from mosquito larvae of the genera *Aedes* and *Culex*. Mosquito larvae of *Anopheles* spp. were placed in sterile plastic cups filled with water and maintained at a relative humidity of 75 ± 5% and a temperature of 28 ± 3 °C in our laboratory. They were fed with commercially available flakes of fish food (Tetramin™). Adult mosquitoes that emerged from the pupae were transferred to a cage and fed with 10% sugar solution. Prior to exposure to insecticides, adult mosquitoes were randomly collected from cages and identified morphologically as *An. gambiae* (*s.l.*). Insecticide susceptibility tests were performed on unfed adult females aged from 3 to 5 days. After 24 h, species identification of all dead mosquitoes and knocked-down surviving mosquitoes by PCR confirmed that they were all *An. gambiae* (*s.l.*).

### Insecticide susceptibility tests

Bioassays were performed using the WHO protocol for adult mosquitoes [32]. Quality-controlled (ISO 9001:2015 certified) impregnated papers were prepared according to the standardized WHO procedures using analytical grade of PESTANAL® insecticide solutions (Sigma-Aldrich, Saint-Quentin Fallavier, France) and validated using insecticide-susceptible Kisumu strain of *An. gambiae* at the laboratory of Maladies Infectieuses et Vecteurs: Ecologie, Génétique, Evolution et Contrôle (MIVEGEC, Institut de Recherche pour le Développement, Montpellier, France). Four different insecticides representing three of four chemical classes available for public health applications against adult mosquitoes were used at the following discriminating concentrations: pyrethroids permethrin (0.75%) and deltamethrin (0.05%), organophosphate malathion (5%) and carbamate bendiocarb (0.1%). WHO tube tests were performed with batches of 20–25 unfed female *An. gambiae* (*s.l.*) mosquitoes aged 3–5 days by exposing them to insecticide-impregnated paper for one hour at 25 ± 2 °C and 70–80% relative humidity. The number of knocked down mosquitoes was recorded at 10, 15, 20, 30, 40, 50 and 60 min before mosquitoes were transferred into holding tubes and provided with cotton wool soaked with 10% sucrose solution. Mortalities were recorded 24 h post-exposure. Batches of 50 mosquitoes exposed to paper impregnated with acetone/olive oil (for bendiocarb and malathion) and acetone/silicone (for permethrin and deltamethrin) were used as control groups.

For each insecticide, dead and live mosquitoes and mosquitoes in the control group were kept separately in 1.5 ml microtubes over silica gel for molecular analysis. The results of the insecticide susceptibility test were validated if mortality in the control group was less than 5% and excluded if the mortality in the control group was more than 20%. The mortality rate between 5 and 20% was corrected using Abbott’s formula [33].

### DNA extraction, species identification and detection of *kdr* mutations

Sub-samples of surviving female mosquitoes (alive after 24 h exposure) and control mosquitoes were randomly selected for molecular analysis. Genomic DNA was extracted from single mosquitoes following the cetyl trimethylammonium bromide (CTAB) protocol described by Le Goff et al. [34], re-suspended in 200 μl of 1× tris-ethylene diamine tetra acetic acid (EDTA) buffer and stored at -20 °C. *Anopheles gambiae* (*s.l.*) species-specific identification was performed in 250 randomly selected specimens using the short interspersed elements (SINE) PCR described by Santolamazza et al. [35], which allows simultaneous discrimination of all members of the *An. gambiae* complex. The West and East African *kdr* mutations in *An. arabiensis* populations were genotyped using the intentional mismatch primer method described by MR4 staff and Huynh [36]. For mosquito genotyping the following allelic designation were used: Rw, west resistant allele; Re, east resistant allele; and S, susceptible allele.

### Data analysis

WHO criteria were used to evaluate the resistance/susceptibility status of the tested mosquito populations [32]. Mortality rates were compared using a Chi-square test, assuming the normality of data when parametric tests were used. Knockdown times (50% or median knockdown time, KDT_50_; and 90% knockdown time, KDT_90_) were determined using the log-probit regression model. Genotypic frequencies of West (L1014F) and East African (L1014S) *kdr* mutations in mosquito population were compared to Hardy-Weinberg expectations using Pearson’s chi-square test. Statistical analyses were performed using MedCalc software (Mariakerke, Belgium) [37]. A probability value of 0.05 or less was considered as significant.

## Results

### Knockdown time

The knockdown times were assessed for permethrin 0.75% and deltamethrin 0.05% in 2015 and 2016. In 2015, 50 and 95% of the specimens of *An. arabiensis* exposed to permethrin 0.75% were knocked down after 66 min (KDT_50_ ranging 53–79 min) and after 244 min (KDT_95_ ranging 231–258 min), respectively. Comparatively, both the KDT_50_ and the KDT_95_ of the study population were shorter with deltamethrin at 39 min (26–52 min) and 119 min (106–132 min), respectively (Table 1). In 2016, the knockdown times of 50 and 95% almost doubled for both insecticides (Table 1).

**Table 1:** Knockdown times of *An. arabiensis* populations exposed to deltamethrin and permethrin in Nouakchott

Insecticide	Year	No. of mosquitoes tested	KDT_50_ (range) in min	KDT_95_ (range) in min
Permethrin (0.75%)	2015	181	66 (53–79)	244 (231–258)
	2016	203	117 (104–131)	489 (475–502)
Deltamethrin (0.05%)	2015	197	39 (26–52)	119 (106–132)
	2016	189	78 (65–91)	257 (244–270)

*Abbreviations*: *KDT*_*50*_ 50% knockdown time, *KDT*_*95*_ 95% knockdown time

### Twenty-four hours post-exposure mortality

Overall, 1494 *An. gambiae* (*s.l.*) were tested for insecticide resistance (702 in 2015 and 792 in 2016). The mortality rate in the control group was less than 5% for all the tests. Therefore, no corrections were required in the test groups. The bioassay showed that *An. gambiae* (*s.l.*) populations from both study sites were highly resistant to permethrin (0.75%) and deltamethrin (0.05%), with mortality rates of 51.9 and 83.7% in 2015, and 24.1 and 39.1% in 2016, respectively (Table 2). The mortality rate in 2016 for permethrin was significantly lower than that observed in 2015 (*χ*^2^ = 31.5, *df* = 1, *P* < 0.001). A similar trend was observed for deltamethrin (*χ*^2^ = 81.1, *df* = 1, *P* < 0.001). The overall mortality rate associated with deltamethrin exposure was significantly higher than that for permethrin (62 *vs* 37.2%; *χ*^2^ = 47.3, *df* = 1, *P* < 0.001). All studied populations of *An. gambiae* (*s.l.*) were fully susceptible (100% mortality) to malathion (2%) and bendiocarb (0.1%) in both 2015 and 2016.

**Table 2:** Mortality rates of female *An. arabiensis* after 24 h exposure to four insecticides in 2015 and 2016 in Nouakchott

Insecticide	2015		2016		*χ* ^2^	*P*
	*n* (# replicate)	Mean mortality (%) (95% CI)	*n* (# replicate)	Mean mortality (%) (95% CI)		
Permethrin (0.75%)	181 (9)	51.9 (44.7–59.1)	203 (10)	24.1 (18.8–30.5)	31.5	< 0.001
Deltamethrin (0.05%)	197 (8)	83.7 (77.9–88.2)	189 (9)	39.1 (32.5–46.3)	81.1	< 0.001
Bendiocarb (0.1%)	106 (5)	100	203 (10)	100	–	–
Malathion (2%)	218 (10)	100	197 (8)	100	–	–

*Abbreviations*: *n* total number of mosquitoes tested, *CI* confidence interval

### Species identification and *kdr* genotyping

**Fig. 2:**
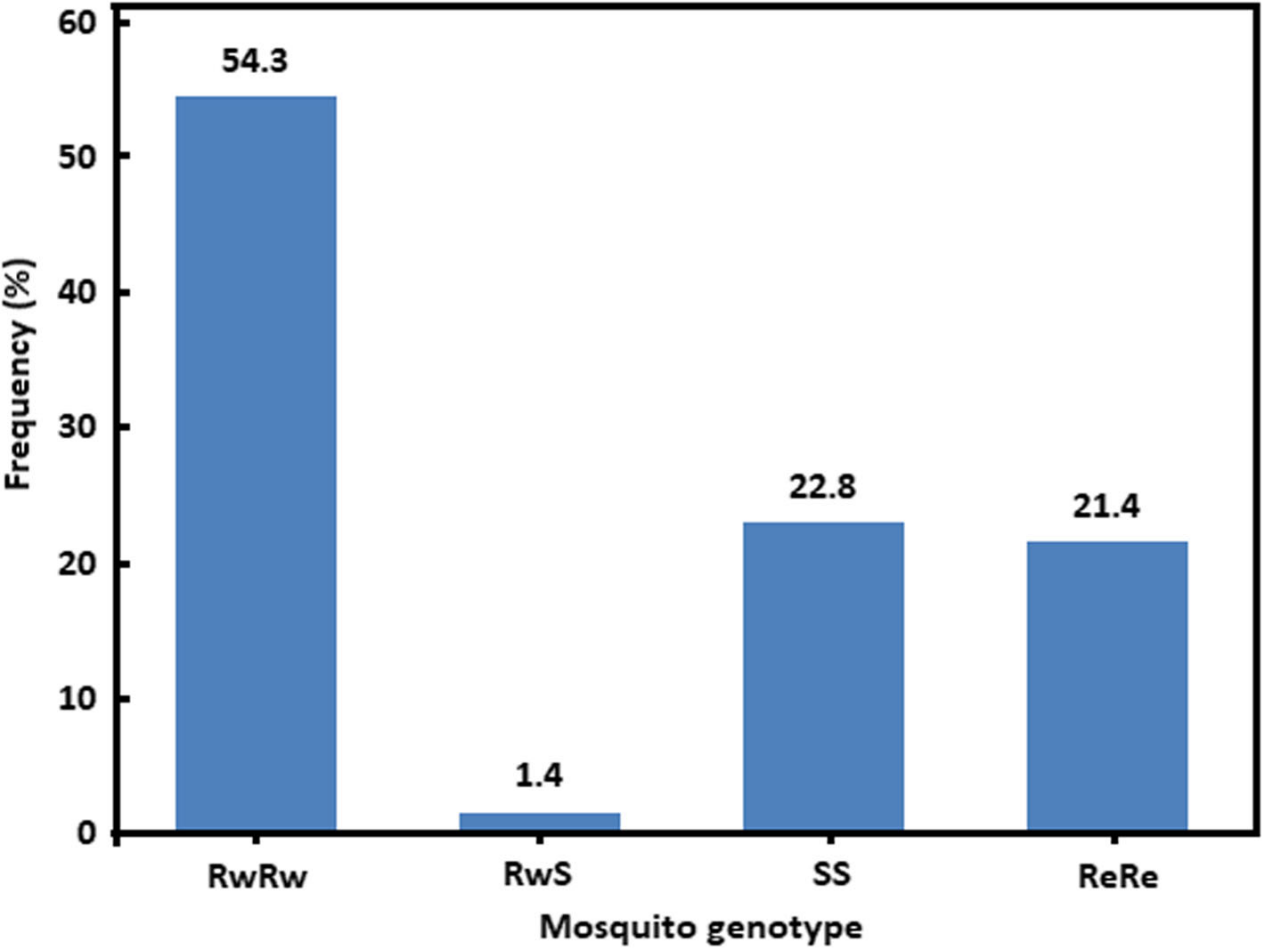
Distribution of genotypic frequencies of *kdr* mutations among *An. arabiensis* mosquitoes surviving insecticide exposure. *Abbreviations*: RwRw, west homozygous resistant; RwS, west heterozygous resistant; SS, homozygous susceptible; ReRe, east homozygous resistant

Of 250 specimens confirmed to be *An. arabiensis* by molecular identification, 70 that survived exposure to insecticides and 15 control female mosquitoes were genotyped for *kdr* mutations. Both West and East African *kdr* resistant alleles were found among the tested specimens. The wild-type and three of five expected *kdr* genotypes were observed among *An. arabiensis* mosquitoes that survived exposure to insecticides (Fig. 2). Heterozygous resistant east-west (ReRw) and heterozygous resistant east (ReS) genotypes were not observed. The homozygous resistant west (RwRw) was the most frequent genotype (54.3%, 38/70), followed by the wild-type homozygous susceptible (SS, 22.8%, 16/70) and the homozygous resistant east (ReRe, 21.4%, 15/70). Only one mosquito (1/70, 1.4%) was heterozygous (RwS) for the West African *kdr* mutation. Nevertheless, the comparison of the observed and expected genotypic frequencies showed a significant departure from Hardy-Weinberg proportions (*χ*^2^ = 134, *df* = 3, *P* < 0.001). This is likely associated with a deficit in heterozygotes as shown by an inbreeding coefficient (*F*_*is*_) of 0.97 according to Weir & Cockerham [38].

## Discussion

Previous entomological studies highlighted the widespread presence of *An. arabiensis* in Mauritania [28]. The present study confirmed that *An. arabiensis* is the main malaria vector in Nouakchott and updated data on its susceptibility to four main insecticides used in public health programs. Our results showed that *An. arabiensis* populations are highly resistant to pyrethroids (permethrin and deltamethrin) but fully susceptible to malathion and bendiocarb in both sites during the study period. This finding is in agreement with the increasing selective pressure on mosquito populations due to the scaling-up of LLINs in the country (National Malaria Control Program, unpublished data), especially in urban areas such as Nouakchott. In Senegal, Niang et al. [39] have reported a dissimilar trend of knockdown dynamic between deltamethrin and permethrin, which they attributed to the more recent use of deltamethrin in comparison with permethrin in their study area, in addition to the known chemical difference of the two molecules (type I *vs* type II) [39]. These authors have hypothesized that the introduction of bednets impregnated with deltamethrin (Olyset Net^©^) in the study area in Senegal in the 1990s could have exerted strong insecticidal pressures on mosquitoes, giving rise to high levels of insecticide resistance. However, a previous study on a knockdown resistance-free *An. arabiensis* population from Chad in Central Africa has reported a partial pyrethroid resistance (resistance to permethrin but not to deltamethrin) in *An. arabiensis* field populations involving metabolic mechanisms of resistance with over-expressed P450 CYP6P4 [40]. By contrast, in Senegalese and Mauritanian *Anopheles* populations, the *kdr*-mediated target site resistance seems to be the main mechanism involved. However, the involvement of metabolic mechanisms of resistance could not be ruled out since 22.8% (16/70) of individuals characterized by the presence of *kdr* mutations did not harbor any of the mutant alleles despite being fully resistant to pyrethroids. The same situation has been previously reported in some malaria vector populations from Senegal [39]. Multiple mechanisms of insecticide resistance in *Anopheles gambiae* (*s.l.*) populations have also been reported elsewhere in *An. arabiensis* [41]. There is a need to assess the potential involvement of metabolic mechanisms of resistance in west African populations of *An. arabiensis*, especially in the Mauritanian population, to explain the increase in the survival rate of *An. arabiensis* populations exposed to pyrethroids in 2016 (mortality rate for permethrin = 24.1% and deltamethrin = 39.1%), compared to 2015 (mortality rate for permethrin = 51.9% and deltamethrin = 83.7%).

Both the West and East African *kdr* mutations were found in the two study sites in Nouakchott, with the West African *kdr* being significantly more prevalent. This finding is in agreement with the general distribution pattern and frequencies of *kdr* mutations in West Africa as shown in the distribution maps of pyrethroid-resistance and underlying mechanisms in African malaria vector [6]. However, a previous study in Nouakchott reported only the presence of the East African *kdr* allele in *An. arabiensis* populations in 2009–2010 [28]. The presence of East African *kdr* allele among *An. gambiae* complex, particularly *An. arabiensis*, is increasingly being observed in several West African countries, such as Benin [42], Burkina Faso [43] and Senegal [44]. The analysis of observed genotypic frequencies revealed a heterozygote deficit for the West African *kdr* allele. Similar data were reported for *An. gambiae* (*s.l.*) from many sites in Burkina Faso [45]. As suggested by several authors, exposure to insecticides used in agriculture, forest exploitation and public health purposes may confer selective advantages to resistant homozygote individuals because *kdr* mutations are a recessive trait [46]. In Nouakchott, the widespread use of pyrethroids within households and for public health programs (LLIN mass distribution campaigns) may induce selection of resistant mosquitoes. The evidence for resistance in *An. arabiensis* to two pyrethroid insecticides (deltamethrin and permethrin) in Nouakchott is of concern and may become an obstacle to successful malaria vector control.

Although the present study is highly informative, there is clearly a need to carry out further studies with larger populations of mosquitoes from different breeding sites, and with more insecticides, to establish a clearer picture of resistance and its molecular basis. Nevertheless, the results of this study should alert health policy makers to the alarming situation of insecticide resistance in *An. arabiensis*, the major malaria vector in Nouakchott where more than one-fourth of the total population of Mauritania resides.

## Conclusions

The study showed a high level of pyrethroid resistance in *An. arabiensis* populations in Nouakchott with a significant increase in the frequency of mutant *kdr* compared to what was observed earlier in Nouakchott. These results stress the need for routine monitoring of the resistance status of malaria vector populations to insecticides in use in the public health sector.

## Abbreviations

*s.l.*: *sensu lato*
*kdr*: Knockdown resistance
PSC: Pyrethrum space-spray catch
WHO: World Health Organization
LLINs: Long-lasting insecticide-treated nets
IRS: Indoor residual spraying
Rw: West resistant alleles
Re: East resistant allele
S: Susceptible allele
